# Treatment of slow transit constipation-induced ileus during pregnancy by colectomy with ileorectal anastomosis: A case report

**DOI:** 10.1097/MD.0000000000019944

**Published:** 2020-05-01

**Authors:** Rui Wang, Qi Su, Zhaopeng Yan

**Affiliations:** a Department of Critical Care Medicine; b Department of General Surgery, Shengjing Hospital of China Medical University, Shenyang, Liaoning, China.

**Keywords:** colectomy, pregnancy, slow transit constipation

## Abstract

**Introduction:**

Slow transit constipation is a major cause of chronic constipation. During pregnancy, changes in hormone levels and the physical effects of an enlarged uterus could cause new onset slow transit constipation or aggravate a pre-existing constipation. The management of slow transit constipation-induced ileus during pregnancy is a medical dilemma.

**Patient concerns:**

A 28-year-old pregnant woman presented to the emergency department with a 7-day history of worsening bloating and abdominal colic. The patient was in her third trimester (27 weeks). She had a 5-year history of constipation which had worsened with her pregnancy, and neither flatus nor stool could be passed.

**Diagnosis:**

Based on the constipation history and computed tomography, a slow transit constipation-induced ileus was confirmed.

**Interventions:**

As medications for the management of constipation and endoscopic efforts to remove the blockage were ineffective and the patient's symptoms worsened, Cesarean section and colectomy with ileorectal anastomosis were performed.

**Outcomes:**

After the procedure, the patient recovered and defecated well. At the 6-month follow-up, the patient reported that she defecated two to three times per day without difficulty.

**Conclusion:**

Pregnancy can worsen pre-existing constipation and cause ileus. In cases where drug treatment is unsuccessful, colectomy, and ileorectal anastomosis may be necessary.

## 1 Introduction

Constipation is a very common symptom.^[1–3]^ Slow transit constipation is a major subtype of chronic constipation in adults.^[4]^ The management of slow transit constipation includes an increase in dietary fiber intake, laxatives, and intestinal prokinetic agents.^[5–9]^ For severe cases such as low transit constipation-induced ileus, an emergent surgical procedure is indicated. During pregnancy, changes in hormone levels and the physical effects of an enlarged uterus could cause new onset slow transit constipation or aggravate pre-existing constipation.^[10,11]^ Usually, fiber supplementation, stimulant laxatives, and bulk-forming laxatives work well for constipation during pregnancy. However, when slow transit constipation is severe enough to cause ileus, treatment can threaten both the mother and fetus. We have found no published reports of colectomy with ileorectal anastomosis performed during pregnancy to treat slow transit constipation-induced ileus. Here, we present a case of successful surgical treatment for slow transit constipation-induced ileus during pregnancy by colectomy with ileorectal anastomosis.

## 2 Case presentation

**Figure 1:**
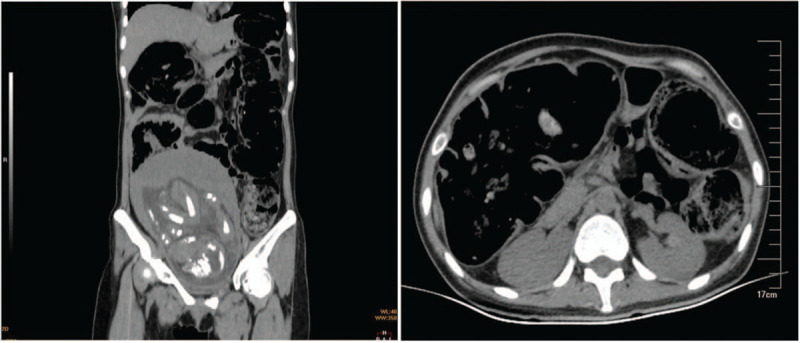
Pre-surgery CT scan. The colon is distended.

A 28-year-old pregnant woman presented to the emergency department with a 7-day history of worsening bloating and abdominal colic. This was her second pregnancy and there were no signs of labor. The patient was in her third trimester (27 weeks). She had a 5-year history of constipation with use of osmotic laxatives, resulting in bowel movements occurring every 3 to 4 days. The stool was hard and lacked water. Her constipation worsened during her pregnancy, and neither flatus nor stool could be passed. Based on her history, the patient was suspected to have slow transit constipation-induced ileus. After administration of osmotic laxatives and prucalopride, the patient passed some flatus, but the colic and bloating had worsened. As her abdominal pain became persistent and more severe, a computed tomography (CT) scan was performed, revealing diffuse distension of the colorectum and a fecalith stuck in the sigmoid colon (Fig. 1). We performed an endoscopy and attempted to fragment the stone, but the procedure was unsuccessful. As all of the conservative treatments had failed, a surgical procedure was considered necessary. After the obstetrician completed a Cesarean section, we performed a colectomy and ileorectal anastomosis.

**Figure 2:**
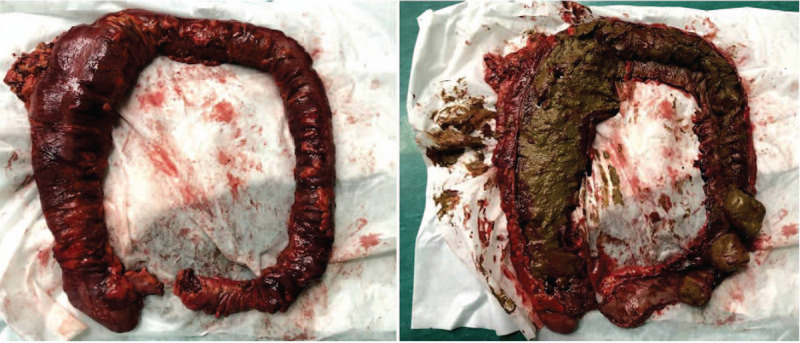
The excised colon. The entire colon is filled with stool, which is harder toward the distal colon.

Upon assessment of the specimen, we found that the entire colon was filled with stool, which was harder toward the distal colon (Fig. 2). The stool in the sigmoid colon was even harder than metal, and lacerations were visible in the serous and muscular layers of the sigmoid colon. Interestingly, the CT scan 8 h prior to surgery revealed that the colon appeared to be full of gas (Fig. 1). However, based on the surgical findings, the CT scan actually showed gas that had fully enclosed the solid stool.

After the procedure, the patient recovered well and was discharged. At the 6-month follow-up, the patient reported that she defecated two to three times per day without difficulty, and the shape and appearance of the stool were normal.

## 3 Discussion

Chronic constipation is a common colorectal disease that brings much pain to patients. Slow transit constipation is an important subtype of chronic constipation. The management of slow transit constipation includes an increase in dietary fiber intake, over-the-counter laxatives, and some new drugs such as linaclotide and prucalopride.^[5–9]^ For those who have not responded well to drug treatment, a colectomy with ileorectal anastomosis is the most common surgery performed in patients with slow transit constipation.^[12,13]^

Constipation is a common symptom experienced during pregnancy. Fiber supplementation, stimulant laxatives, and bulk-forming laxatives are usually adequate to manage constipation during pregnancy.^[9]^ Although postpartum dynamic ileus has been widely reported, reports of dynamic ileus during pregnancy are rare. In a retrospective study in a single medical center, there were 7 cases of dynamic postpartum ileus among 10,240 pregnancies in 2 years. Only one of these seven cases needed an emergent surgical procedure, and a successful right hemicolectomy with anastomosis and creation of a defunctioning loop ileostomy was performed.^[14]^

In this case, a woman presented with aggravated constipation during pregnancy, subsequently resulting in ileus. After 1 week of conservative treatments, her symptoms and abdominal signs deteriorated. Considering the potential threats to the woman and the developing fetus, we performed surgery. Upon exploration, her entire colon was distended and full of stool. The haustra of the colon had disappeared, and several hard stools were stuck in the sigmoid. Due to extensive stool compaction, the sigmoid colon had stretched and was dark red in appearance, with tears in the serous and muscular layers, indicating potential risk for perforation. As the ileocecal valve function was normal, and the stool in the large bowel did not transmit backward into the small intestine, the small intestinal was not distended and had no edema or inflammation. Her rectum was also normal, with no obstruction or anatomical abnormalities observed. Considering that the intestine and rectum were normal, we made an ileorectal anastomosis, and confirmed the absence of anastomotic stoma leakage.

During pregnancy, changes in the immune, hormonal, and nutritional status make the post-operative recovery process more complicated.^[13]^ Furthermore, the enlargement of the uterus often compresses the anastomotic stoma, which increases pressure on the colon, thereby increasing the risk for anastomotic leakage. Our patient was immediately postpartum at the time of surgery, and therefore at higher risk for pelvic infection, hyper-coagulation, and thrombotic events.^[15]^ Abdominal colectomy and ileorectal anastomosis are indicated for cases like ours involving slow transit constipation without outlet obstruction or dyssynergic defecation.^[16,17]^ Our patient recovered well despite the additional, pregnancy-related risks.

## 4 Conclusion

Slow transit constipation is a common colorectal disorder. Pregnancy can worsen pre-existing constipation and in rare cases can cause ileus. In cases where drug treatment is unsuccessful, colectomy and ileorectal anastomosis may be an effective and safe option.

## Author contributions

**Data curation:** Rui Wang, Qi Su, Zhaopeng Yan.

**Writing – original draft:** Rui Wang.

**Writing – review & editing:** Zhaopeng Yan.

Zhaopeng Yan orcid: 0000-0003-1247-2067.
